# Decreased Expression of Karyopherin-α 1 is Related to the Malignant Degree of Cervical Cancer and is Critical for the Proliferation of Hela Cells

**DOI:** 10.3389/pore.2022.1610518

**Published:** 2022-08-04

**Authors:** Lucen Jiang, Dan Li, Chao Wang, Jia Liao, Jianghuan Liu, Qingzhu Wei, Yiyang Wang

**Affiliations:** ^1^ Department of Pathology, The Third Affiliated Hospital of Southern Medical University, Guangzhou, China; ^2^ Department of Pathology, The Sixth Affiliated Hospital of Sun Yat-Sen University, Guangzhou, China; ^3^ Department of Pathophysiology, School of Medicine, Jinan University, Guangzhou, China

**Keywords:** cervical cancer, proliferation, KPNA1, Hela cell, malignant

## Abstract

Karyopherin α (KPNA) proteins are involved in nucleocytoplasmic trafficking and are critical for protein subcellular localization. Recent studies have suggested that KPNA proteins are abnormally expressed in various solid tumors. The objective of this study was to investigate the expression of KPNA1 and KPNA2 in cervical cancer tissue with different histologic grades and cell lines, as well as the effects of the KPNA1 expression level on Hela cell proliferation. We collected the medical data of 106 patients with cervical cancer and investigated the protein expression of KPNA1 and KPNA2 by immunohistochemistry and western blot. The results revealed a significantly lower expression of KPNA1 in cervical cancer compared to normal tissue. Conversely, stronger staining intensity for KPNA2 was observed in cervical tumor samples. The expression levels of KPNA1 and KPNA2 were significantly associated with the tumor histologic grade. The weakest KPNA1 expression and strongest staining for KPNA2 were observed in grade III tumor tissue. The expression levels of KPNA1 were lower in Hela and C33A cells compared with normal human cervical epithelial cells; however, the expression of KPNA2 exhibited an opposite trend. The up-regulation of KPNA1 significantly suppressed the proliferation of Hela cells and relevant proteins expression, as well as promoted transportation of IRF3 into nucleus. Our results suggest the downregulation of KPNA1 expression is related to the malignant degree of cervical cancer and is closely associated with the proliferation of cervical cancer cells.

## Introduction

Despite progress in screening and the development of a vaccine, cervical cancer is the second most common gynecologic malignancy and the third leading cause of cancer deaths among women in developing countries. Approximately 604,127 new cases of cervical cancer and 341,831 deaths were reported worldwide in 2020. In total, 84% of the new cases and 87–90% of the deaths occurred in less-developed regions (1, 2). In 2018, the age-standardized incidence rate of cervical cancer among Chinese females was still as high as 10.7% (3). The therapeutic strategies for cervical cancer patients vary substantially with histological subtype, malignancy grade, patient age, preoperative performance score, etc. Currently, early-stage cervical cancer is treated with a radical hysterectomy operation, whereas chemotherapy concurrent with radiation therapy is the treatment of choice for the advanced stages (4, 5). It has been demonstrated that about 95% of invasive cervical cancer is caused by persistent infection with human papillomavirus (HPV), including cervical squamous cell carcinoma and adenocarcinoma (6, 7). Pap smears and HPV co-testing can be used to effectively screen for cervical cancer (8), however, these cannot be used as diagnostic bases for distinguishing different malignant grades of cervical cancer. Current diagnoses are very dependent on tumor histology. In addition to traditional morphological analysis, immunohistochemical and molecular biological biomarkers may enable further clarification of the diagnosis.

Karyopherin proteins are involved in the nucleocytoplasmic trafficking of cargo proteins and certain RNAs, across the nuclear pore complex into and out of the cell nucleus. As an adaptor protein, karyopherin α (KPNA) mediates the nuclear translocation of numerous target proteins through the nuclear pore complex *via* interacting with the nuclear localization signal (9, 10). It has also been shown to play a key role in the cell cycle, mitosis and replication (11). A number of studies have reported that KPNA2, a member of the karyopherin α family, is overexpressed in multiple forms of cancer, including breast cancer, lung cancer, esophageal squamous cell carcinoma, colon cancer, prostate cancer and cervical cancer (12–16). Recently, our preliminary results demonstrated that KPNA2 immunohistochemical expression was highly sensitive and specific for osteosarcoma (17). The expression of KPNA2 is also reportedly associated with poor prognosis in patients with gastric cancer, epithelial ovarian carcinoma and small hepatocellular carcinoma (18–20). However, KPNA1, another karyopherin α family member, has been poorly researched in tumors.

The purpose of the present study was to evaluate the expression levels of KPNA1 in cervical cancer samples of patients using immunohistochemistry and confirm their potential diagnostic utility as a novel molecular marker for discriminating among different malignant grades of cervical cancer. Furthermore, the effects of KPNA1 on the proliferation of Hela cells were investigated.

## Materials and Methods

### Materials

The pCMVTNT-T7-KPNA1 (#26677) plasmid was obtained from Addgene. Anti-PCNA (sc-56), anti-Cyclin D1 (sc-8396), anti-IRF3 (sc-33641), anti-KPNA1 (sc-101292), anti-KPNA2 (sc-55538) antibodies and KPNA2 siRNA (sc-35741) were purchased from Santa Cruz Biotechnology, Inc. (Santa Cruz, CA, United States). Antibody against GAPDH (#2118) was purchased from Cell Signaling Technology, Inc. (Beverly, MA, United States). Lipofectamine 2000 (#11668019), Goat anti-mouse IgG antibody (#31438) and Fluor 488-labelled goat antibody against mouse IgG (A32723) were obtained from Thermo Fisher Scientific (Logan, UT, United States).

### Patients and Specimens

This study was approved by the Ethical Committee of The Third Affiliated Hospital of Southern Medical University. Before implementation, patients agreed to the use of the samples in scientific research and signed the informed consent form. Since most cervical cancer patients have squamous cell carcinoma, we chose this histological subtype of cervical cancer as the research object. In total, 106 cervical cancer tissue samples were evaluated for the expression of KPNA1 and KPNA2. Normal tissue specimens adjacent to the cancerous tissue served as controls. All the tissue samples accessioned between January 2016 and March 2021 were retrieved from the surgical pathology and consultation files of the Third Affiliated Hospital of Southern Medical University in China. The diagnosis of a malignant tumor was established based on the tumor location, its histomorphology, and/or the results of immunohistochemical studies. All the biopsy slides were examined independently by two experienced pathologists (Lucen Jiang and Dan Li). The patients were staged by clinical examination according to the International Federation of Gynecology and Obstetrics (FIGO) criteria (21). The histopathologic grades are as follows: Grade I, well differentiated; Grade II, moderately differentiated; Grade III, poorly differentiated. The clinicopathological features of the patients with cervical cancer are presented in Table 1.

**TABLE 1:** Clinicopathological features of the study population.

Characteristic	Patients (*n* = 106)
Age (Years), median (minimum–maximum)	52 (25–80)
Tumor source, n (%)	
Biopsies	5 (4.7)
Hysterectomy	101 (95.3)
Tumor size, n (%)	
2 cm or less	39 (36.8)
More than 2 cm	67 (63.2)
FIGO stage, n (%)	
I	72 (67.9)
II	8 (7.6)
III	25 (23.6)
IV	1 (0.9)
Pathologic grade, n (%)	
G1	17 (16.0)
G2	80 (75.5)
G3	9 (8.5)
LVSI, n (%)	
Positive	70 (66.0)
Negative	36 (34.0)
Lymph node metastasis, n (%)	
Positive	27 (25.5)
Negative	79 (74.5)

LVSI, lymphovascular space invasion; FIGO, Federation International Gynecologica Obstetrica (International Federation of Gynecology and Obstetrics).

### Immunohistochemical Analysis

All the tumor and normal tissue samples were fixed in formalin. Paraffin-embedded normal control and tumor tissues of patients with cervical cancer were cut into 4 μm-thick sections with standard techniques. Some paraffin sections were used for hematoxylin-and-eosin (H&E) staining. Antibodies against KPNA1 and KPNA2 were used to analyze other sections. The negative control was set up by using mouse IgG instead of primary antibody. Immunohistochemical staining was performed according to the manufacturer’s instructions. Then, the sections were visualized using a microscope (BX45, Olympus Corporation, Tokyo, Japan). The number and intensity of positive cells were determined in five randomly selected fields per section at high magnification. Afterwards, the mean value from five images per chamber was calculated. The extent of staining was scored according to the percentage of positive cells as follows: 1, <5%; 2, 5–25%; 3, 25–50%; 4, 50–75%; 5, >75%. The intensity score was graded as follows: 0, no staining; 1, weak staining; 2, moderate staining; and 3, strong staining. The final score was calculated using the following formula: total score = percentage score × intensity score. The median value of 5% was defined as a cutoff for negative and positive staining. Image J software was used to quantify the signal intensity of staining.

### Cell Culture

The Hela, C33A and human primary cervical epithelial (HCE, PCS-480-011™) cells were obtained from the American Type Culture Collection (ATCC). The Hela and C33A cells were cultured in Dulbecco modified Eagle medium (DMEM) supplemented with 10% fetal bovine serum (FBS; Gibco, Rockville, MD, United States). The HCE cells were cultured in cervical epithelial cell basal medium (ATCC PCS-480-032) with 10% FBS at 37°C in a humidified atmosphere containing 5% CO_2_.

### Immunofluorescence Staining

Hela and HCE cells were fixed with 4% formalin solution. The cells were then treated with 0.1% Triton X 100 in PBS to make intracellular protein binding sites more accessible for the antibodies. After 3 washes with PBS for 5 min each time, the cells were blocked in PBS with 1% BSA, followed by incubation with a 1:100 dilution of antibodies against KPNA1 or KPNA2 at 4°C overnight. The cells were then incubated with the secondary antibodies using Alexa Fluor 488-labelled goat antibody against mouse IgG (1:400 dilution) for 1 h. Finally, after washing three times, the cells were incubated with a 1: 200 dilution of 4’, 6-diamidino-2-phenylindole (DAPI) for 15 min and observed via laser confocal microscopy.

### Western Blotting Analysis

Western blot experiments were performed as previously described (22). The tissues and cells were lysed in RIPA buffer containing a protease inhibitor (PMSF) to harvest total proteins. The extraction of nuclear proteins was performed by using a commercially available Nuclear Extraction kit (#78833, Thermo Fisher Scientific, Logan, UT, United States) according to the manufacturer’s instructions. The proteins were subjected to SDS-PAGE (sodium dodecyl sulfate-polyacrylamide gel electrophoresis) and then electrophoretically transferred to polyvinyl difluoride (PVDF) membranes. Following blockage of nonspecific binding sites with 5% nonfat dry milk for 1 h, the membranes were incubated with the appropriate primary antibodies against KPNA1, KPNA2, GAPDH, PCNA, Cyclin D1, IRF3 and Lamin B1 (1:1000 dilution) overnight at 4°C. After being rinsed with TBST, the membranes were incubated in a 1: 20,000 dilution of horseradish-peroxidase-conjugated secondary antibody (Thermo Fisher Scientific, Logan, UT, United States) for 1 h at room temperature. The immunoblotted proteins were visualized with enhanced chemiluminescence (ECL) reagents. Blots from at least three independent experiments were used for quantification purposes, and representative data are shown. The ImageJ software, an open-source image-processing program, was used to quantify the blots.

### Transfection of KPNA1-Overexpression Plasmid

pCMVTNT-T7-KPNA1 (#26677) was obtained from Addgene. The blank vector was used as a control plasmid. The KPNA1-overexpression and control plasmids were transfected into Hela cells using Lipofectamine 2000, following the manufacturer’s instructions.

### Cell Proliferation Assay

Cell proliferation was quantified using Cell Counting Kit-8 (CCK-8; CK04-11, Dojindo, Kumamoto, Japan) and EDU staining (C10340, Thermo Fisher Scientific, Logan, UT, United States). For the CCK-8 assay, Hela cells were seeded into 96-well plates at a density of 1×10^4^ cells/mL and transfected with pCMVTNT-T7-KPNA1 plasmid for 24, 48, 72 and 96 h. Next, the medium was removed, and the cells were washed twice with PBS. Then, 10 μl of CCK-8 was added to each well, followed by incubation for 2 h at 37°C. The optical density (OD) at 450 nm was detected utilizing an Epoch 2 Microplate Spectrophotometer (BioTek Instruments, Winooski, VT, United States). The experiments were performed in triplicate.

The detection of EdU incorporation into the DNA was performed with the Click-iT EdU Alexa Fluor-647 Cell Proliferation Kit (C10340, Thermo Fisher Scientific, Logan, UT, United States), according to the manufacturer’s instructions. Briefly, cultured Hela cells were plated in 24-well plates at a density of 2 × 10^5^ cells/well and transfected with pCMVTNT-T7-KPNA1 and control plasmid for 48 h before exposure to EdU. After incubation for 2 h, the cells were washed three times with 3% BSA in PBS and then fixed with 4% paraformaldehyde for 10 min. After washing with 3% BSA, the cells were permeabilized with 0.5% Triton X-100 in PBS for 20 min at room temperature. The cells were washed with 3% BSA three times and then incubated with the EdU staining cocktail at room temperature for 30 min, while protected from light. Images were taken under a fluorescence microscope. The proportions of cells positively stained were determined in at least five randomly selected fields from each section.

### Statistical Analysis

All data were expressed as the mean ± standard error of the mean (SEM). Shapiro-Wilk test was used to evaluate the data for normal distribution. Statistical comparisons among groups were made using Student’s t test or one-way ANOVA. Differences with *p* values < 0.05 was considered to be statistically significant. The SPSS software was used to analyze the data.

## Results

### Patient Characteristics

The clinicopathological characteristics of the patients with cervical cancer are shown in Table 1. The clinical data for 106 cervical cancer patients, with a median age of 52 years (range, 25–80 years), were retrieved from hospital data files in 2021. Five of the samples were from colposcopy biopsies and the rest were obtained by hysterectomy. According to FIGO staging, 72 (67.9%) patients were Stage I, 8 (7.6%) patients were Stage II, 25 (23.6%) patients were Stage III, and 1 (0.9%) patient was Stage IV. On the basis of the original histopathologic grades, 16% of the patients had well-differentiated (Grade I) cancers, 75.5% of the patients had moderately differentiated (Grade II) cancers, and 8.5% of patients had poorly differentiated (Grade III) tumors (Table 1).

### Immunohistochemical and Western Blotting Analysis of KPNA1 and KPNA2 in Cervical Cancer Tissues

**FIGURE 1:**
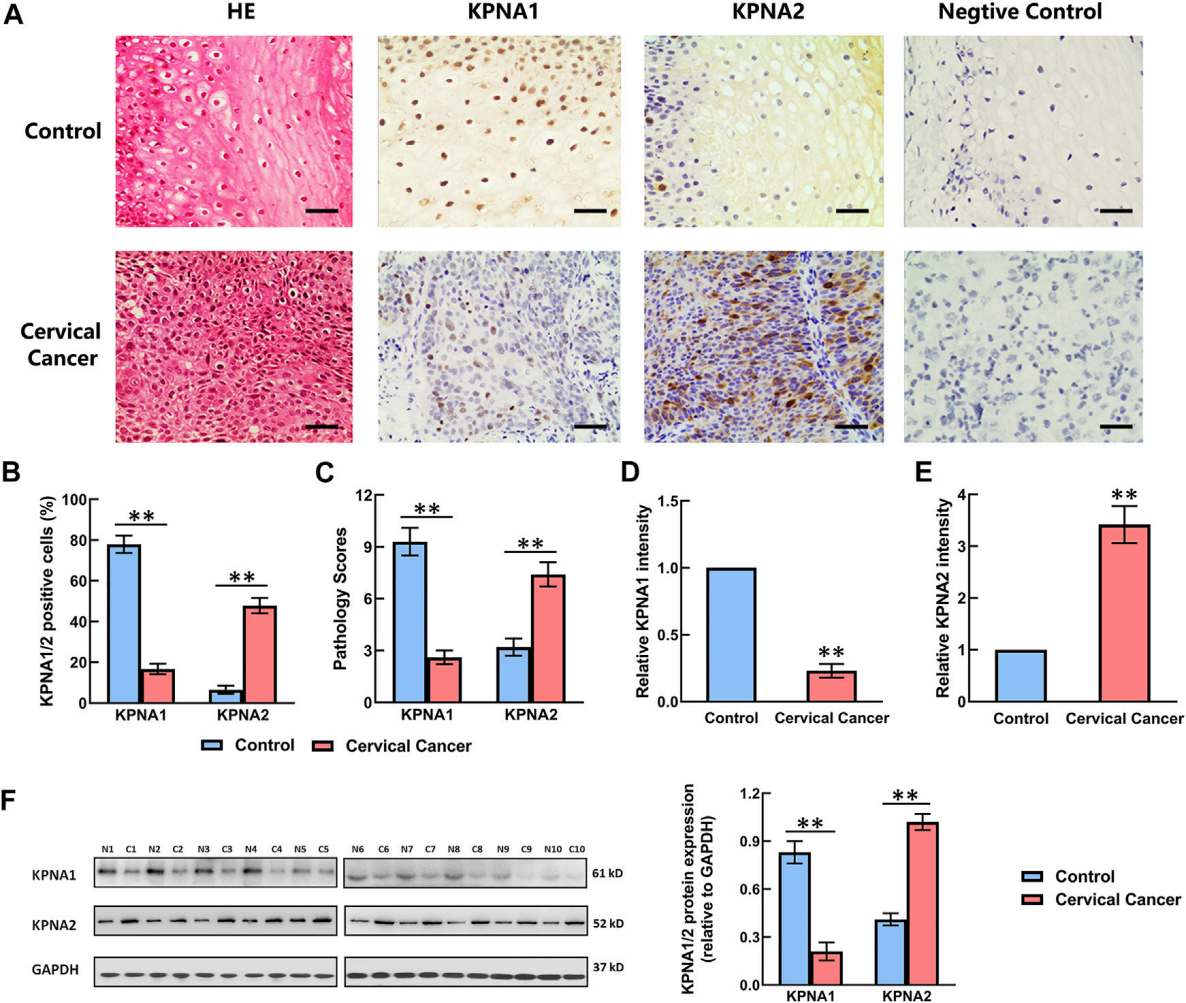
Comparison of the expression levels of KPNA1 and KPNA2 in normal cervical tissues and cervical cancer tissues based on immunohistochemical staining and western blot. **(A)** Representative hematoxylin-and-eosin (HE) staining and antibody staining of the normal and cervical cancer tissues. Scale bar = 200 μm. **(B)** The percentages of KPNA1/2-positive cells were calculated based on five randomly selected fields in the stained normal and cervical cancer tissues. **(C)** The pathology scores for KPNA1/2-positive staining in the normal and cervical cancer tissues. **(D,E)** The relative intensity of KPNA1 and KPNA2 in the stained normal and cervical cancer tissues were quantified by Image J. **(F)** The expressions of KPNA1 and KPNA2 were detected by western blot analyses. Quantification of the relative protein levels is on the right-hand side of the images. N is for normal tissue; C is for cervical cancer tissue. *n* = 10. Values shown are mean ± SEM. **p < 0.01 vs. the control group.

The expression of KPNA1 and KPNA2 in the tumor tissues (*n* = 106) and adjacent tissues was detected by immunohistochemistry. The negative control was set up by using mouse IgG instead of primary antibody. Ten samples were randomly selected to detect the expression of KPNA1 and KPNA2 by western blot. The staining results showed that the immunoreactivity for KPNA1 was significantly lower in the cervical cancer tissues than the normal tissues, whereas KPNA2 displayed the opposite trend, with increasing immunostaining in the cervical cancer tissue over the normal tissue (Figure 1A). We also counted the KPNA1-and KPNA2-positive cells in normal and cervical cancer tissues and calculated the pathological scores associated with KPNA1/2 expression. There was no staining in the negative control. Our analysis confirmed that the number of KPNA1-positive cells, corresponding pathological scores and staining intensity of KPNA1 were obviously lower in cancer tissues compared with normal tissues. By contrast, an elevated expression of KPNA2 in cervical cancer tissues was observed (Figures 1B–E), in line with the literature describing the overexpression of KPNA2 as a biomarker for multiple types of cancer, including cervical cancer. Meanwhile, the western blotting result showed the same trend as immunohistochemical analysis (Figure 1F).

### The Expression of KPNA1 was Downregulated With the Aggravation of Cervical Tumor Malignancy

**FIGURE 2:**
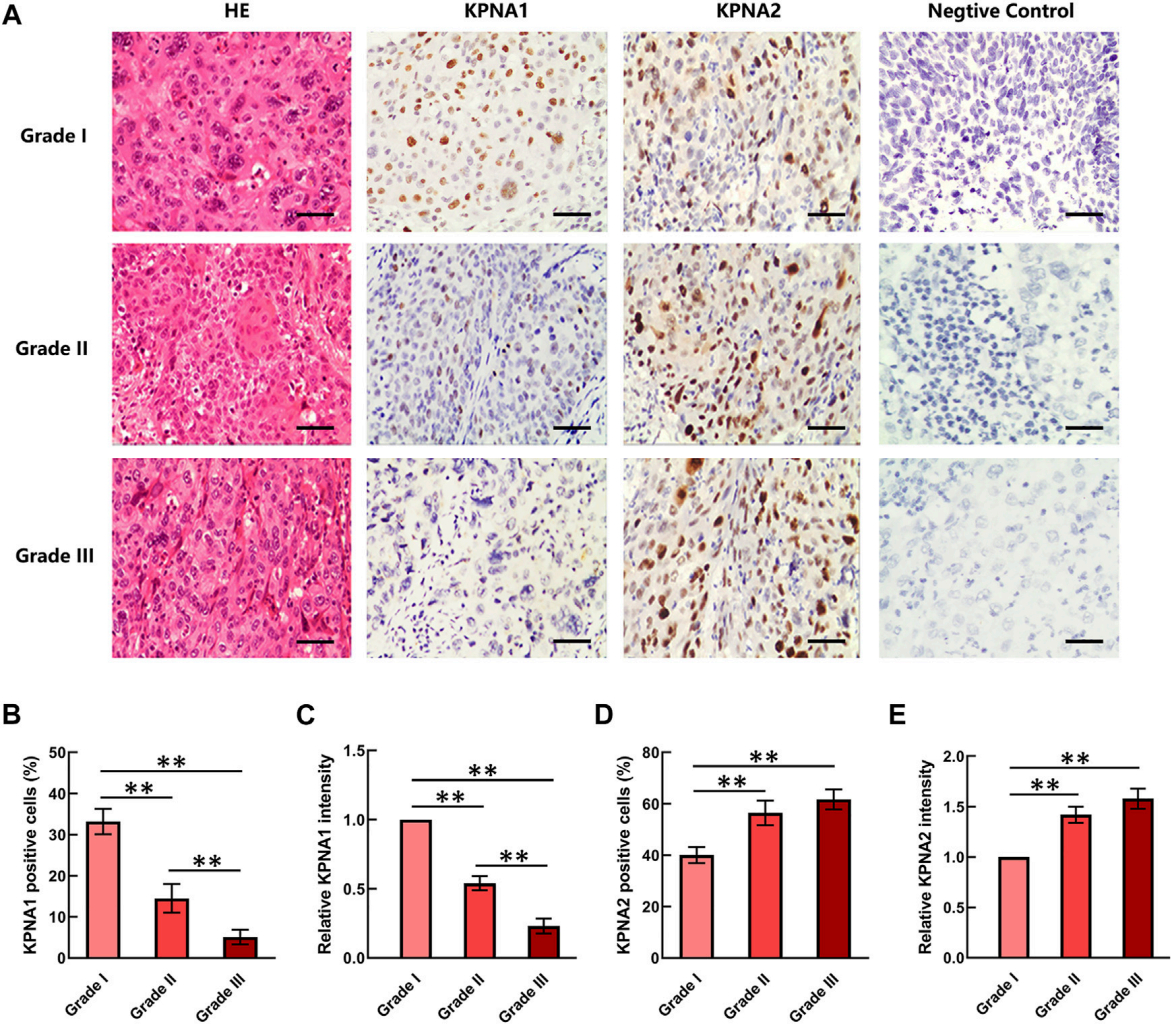
KPNA1 and KPNA2 immunostaining for different grades of cervical malignant tumor tissues. **(A)** Representative HE staining and antibody staining of the different grades of cervical cancer tissues. Scale bar = 200 μm. **(B,C)** The percentages and relative intensity of KPNA1-positive cells were calculated based on five randomly selected fields in the stained cervical cancer tissues. **(D,E)** The percentages and relative intensity of KPNA2-positive cells in the different grades of cervical cancer tissues. Values shown are mean ± SEM. **p < 0.01.

Three grades of cervical cancer are recognized based on the histopathologically differentiated degrees of the tumor: Grade I, Grade II and Grade III. To further confirm the correlation between KPNA1/2 levels and the degree of malignancy of cervical cancer, we further detected the expression of KPNA1 and KPNA2 in cervical tumor tissues with different histopathologic grades by immunohistochemical staining. As shown by the results in Figure 2A, the highest KPNA1 expression and lowest KPNA2 expression were observed in Grade I tumors. Moreover, KPNA1 expression was moderately downregulated and extensively decreased in the Grade II and III cervical cancer specimens, respectively. The percentage of KPNA1-positive cells and the relative intensity in Grade III cancer tissues were markedly lower than those in Grade I and II tissues. Although most of the tumor samples were Grade II, the smallest proportion and weakest intesity for KPNA1 immunostaining were observed in the Grade III samples (Figures 2B,C). The extent and intensity of KPNA1/2 expression, reflecting the immunohistochemical results for the different grades, are summarized in Tables 2, 3. Of the Grade I samples, 12 of 17 (70.6%) were positive for KPNA1, with six (35.3%) samples showing strong-intensity staining. KPNA1 was expressed in 43 of 80 (53.8%) Grade II samples, with 7 (8.8%) samples showing strong-intensity staining. Three of the nine (33.3%) Grade III samples were positive for KPNA2 staining without strong-intensity staining. The least extent and intensity were observed in the Grade III cervical tumor tissues (Table 2). The data confirmed that the downregulation of KPNA1 expression was associated with the degree of malignancy of cervical cancer. However, the proportion of KPNA2-positive staining with strong intensity remained high in all three grades of carcinoma tissues (Figures 2D,E; Table 3).

**TABLE 2:** Extent and intensity of KPNA1 immunohistochemical staining in different pathologic grade of cervical cancer.

Pathologic grade	Positives cases^b^ (%)	Extent of staining^a^						Strong-intensity staining (%)
		0	1+	2+	3+	4+	5+	
Grade I (*n* = 17)	12 (70.6)	2	3	5	4	2	1	6 (35.3)
Grade II (*n* = 80)	43 (53.8)	15	22	26	10	4	3	7 (8.8)
Grade III (*n* = 9)	3 (33.3)	4	2	2	1	0	0	0 (0)
Total (*n* = 106)	58 (54.7)	21	27	33	15	6	4	13 (12.3)

a 0, no staining; 1+, <5%; 2+, 5–25%; 3+, 26–50%; 4+, 51–75%; 5+, 76–100%.

b If it was present in more than 5% of cells, the sample was classified as having positive expression.

**TABLE 3:** Extent and intensity of KPNA2 immunohistochemical staining in different pathologic grade of cervical cancer.

Pathologic grade	Positives cases^b^ (%)	Extent of staining^a^						Strong-intensity staining (%)
		0	1+	2+	3+	4+	5+	
Grade I (*n* = 17)	14 (82.4)	1	2	4	4	3	3	10 (58.8)
Grade II (*n* = 80)	70 (87.5)	3	7	23	26	11	10	57 (71.2)
Grade III (*n* = 9)	8 (88.9)	0	1	1	3	2	2	7 (77.8)
Total (*n* = 106)	92 (86.8)	4	10	28	33	16	15	74 (69.8)

a 0, no staining; 1+, <5%; 2+, 5–25%; 3+, 26–50%; 4+, 51–75%; 5+, 76–100%.

b If it was present in more than 5% of cells, the sample was classified as having positive expression.

### The Levels of KPNA1 and KPNA2 in HCE, Hela and C33A Cells Were Detected by Immunofluorescence and Western Blotting

**FIGURE 3:**
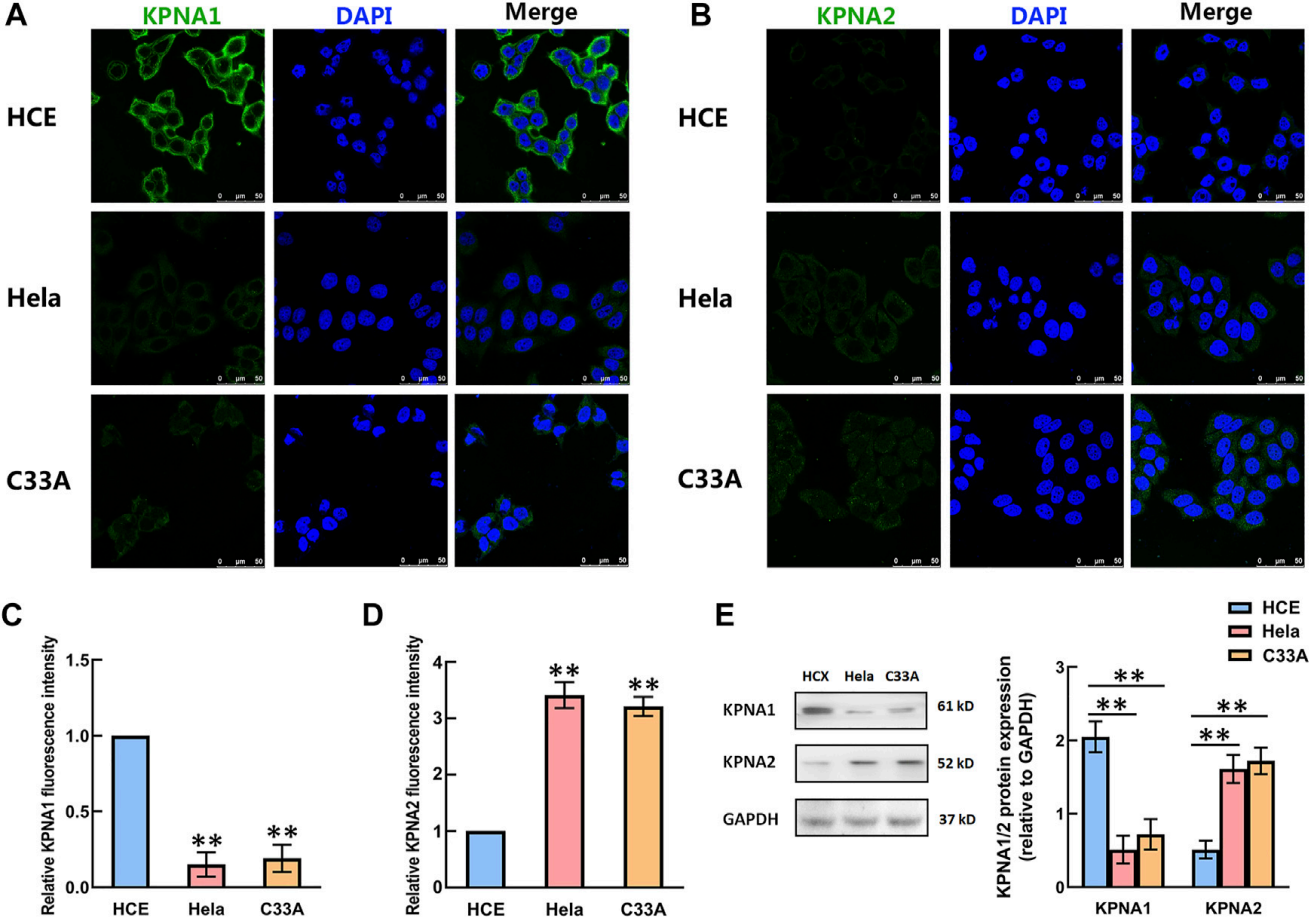
The levels of KPNA1 and KPNA2 in the HCE, Hela and C33A cell lines were detected by immunofluorescence and western blot analyses. **(A,B)** Representative immunofluorescence photomicrographs of KPNA1 and KPNA2 assays (green) in the HCE (upper), Hela (middle) and C33A (bottom) cell lines. Total nuclear staining with DAPI (blue). **(C,D)** The relative KPNA1 and KPNA2 fluorescence intensity of the HCE, and Hela and C33A cell lines. **(E)** The expressions of KPNA1 and KPNA2 were detected by western blot analyses. Quantification of the relative protein levels is on the right-hand side of the images. *n* = 3. **p < 0.01 vs. the HCE group.

We then detected the expression of KPNA1 and KPNA2 in human cervical carcinoma Hela and C33A cells. Meanwhile, HCE (human primary cervical epithelial) cells were chosen as controls. As shown by the results in Figures 3A–D, the immunofluorescent staining of KPNA1 was much stronger in HCE cells than in Hela and C33A cells, whereas the KPNA2 immunostaining was weaker in HCE cells than in Hela and C33A cells. The protein levels of KPNA1/2 in HCE, Hela and C33A cells were further tested by western blot analysis. The results showed that the expression of KPNA1 was markedly lower in Hela and C33A cells than in HCE cells, while KPNA2 protein expression was significantly higher in Hela and C33A cells (Figure 3E). This is consistent with the results of the immunohistochemical analysis in tissues.

### Upregulation of KPNA1 Expression Suppressed the Proliferation of Hela Cells and Increased the Nuclear IRF3 Level

**FIGURE 4:**
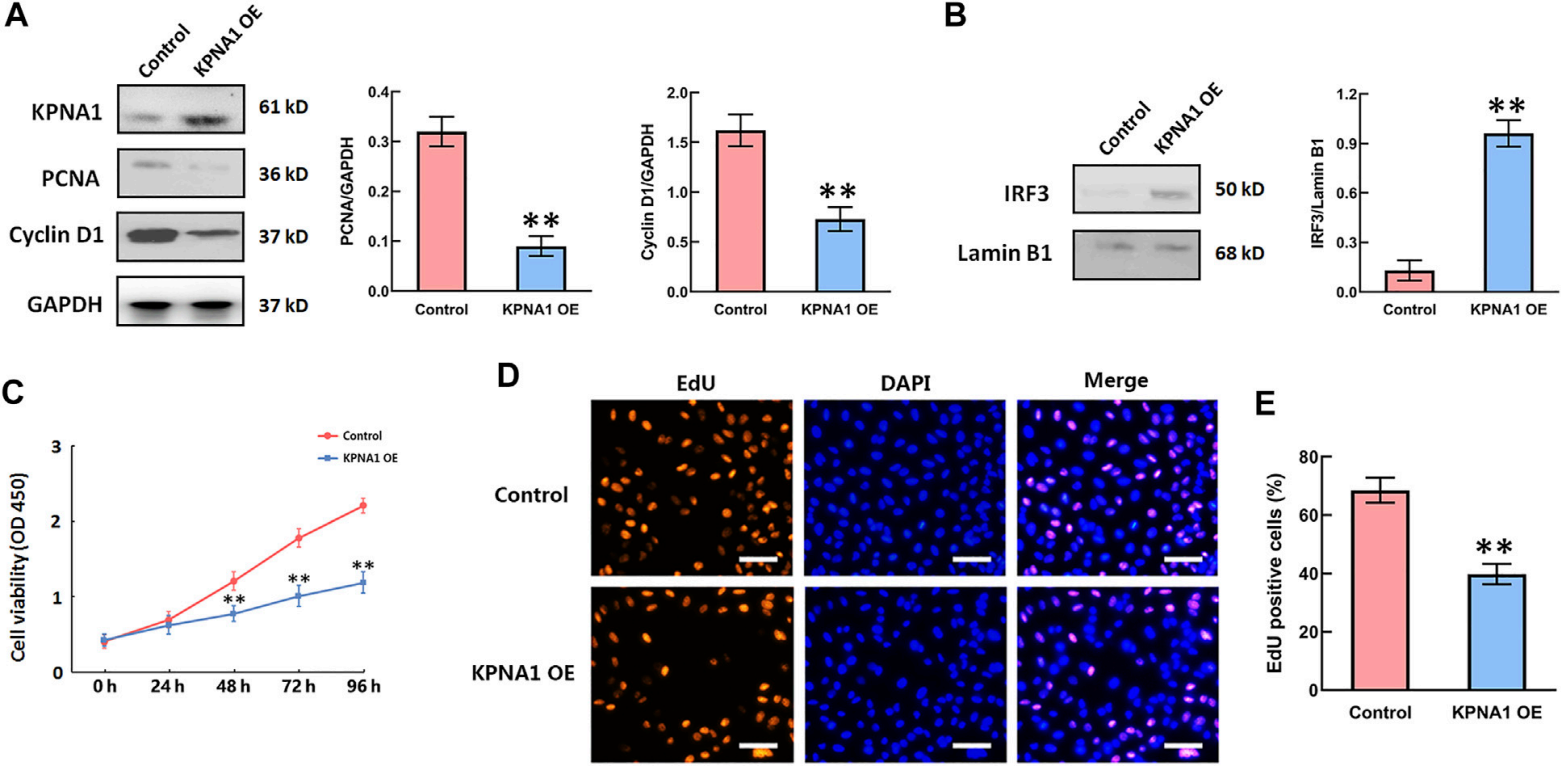
The upregulation of KPNA1 suppressed the proliferation of Hela cells and increased the nuclear IRF3 level. Hela cells were transfected with control and pCMVTNT-T7-KPNA1 plasmids separately. **(A,B)** Western blot detection of KPNA1, PCNA, Cyclin D1 and nuclear IRF3 levels in Hela cells. **(C)** CCK-8 assay detection of Hela cell growth. **(D)** Representative images of EdU staining for cell proliferation. Scale bar = 100 μm. **(E)** Quantification of EdU-positive cells. *n* = 3. **p < 0.01 vs. the control group.

Rapid cell proliferation is a prominent malignant characteristic of cancers. We next sought to determine whether the upregulation of KPNA1 level influenced Hela cells’ proliferation. PCNA is a marker of cell proliferation, cyclin D1 is cell cycle-promoting protein. We overexpressed KPNA1 in Hela cells through pCMVTNT-T7-KPNA1 transfection for 48 h, and then measured the KPNA1, PCNA, Cyclin D1 by western blot. The result sowed that the cell lysates with an anti-KPNA1 antibody revealed higher KPNA1 expression in pCMVTNT-T7-KPNA1-transfected Hela cells than control cells, as well as the expressions of PCNA and Cyclin D1 were significantly decreased after KPNA1 upregulation in Hela cells (Figure 4A). Interferon regulatory factor-3 (IRF3), a transcription factor, plays an essential role in the induction of type I interferons that play an anti-tumor role (23). Yeon, et al reported that IRF-5 is transported to the nucleus by binding to nuclear import proteins KPNA1 and KPNA-β1 (24). On this basis, KPNA1 may promote IRF3 to enter the nucleus. Our data showed that upregulation of KPNA1 obviously increased the level of nuclear IRF3 (Figure 4B). Furthermore, the cell proliferation was detected by using the CCK-8 assay and EdU staining. As shown in Figure 4C, the proliferation of Hela cells was significantly reduced by the overexpression of KPNA1 after plasmid transfection for 48, 72, and 96 h. We also found knockout of KPNA2 inhibited the proliferation of Hela cells (Supplementary Figure S1). The EdU staining results showed that the proportion of positively stained Hela cells was much lower following transfection with the KPNA1-overexpression plasmid than following transfection with the control plasmid (Figures 4D,E). It means that the proliferation of Hela cells was markedly restrained by the upregulation of KPNA1 expression.

## Discussion

Cervical cancer is the fourth most common cancer in women, with an estimated 570,000 new cases in 2018, representing 6.6% of all female cancers worldwide (25). The prognosis for cervical cancer patients varies substantially with the FIGO stage, malignancy grade, patient age and treatment. The current primary treatment for patients with cervical cancer is either surgery or chemoradiotherapy; this is very dependent on the tumor histology. Extending the traditional morphological analysis to include molecular biological, immunohistochemical and genetic biomarkers would be beneficial for the further accurate stratification of patients and, eventually, even individualized therapy. As a member of the karyopherin family for nuclear protein transport, KPNA2 has been observed to show abnormally high expression in cervical cancer patients (16). In this study, we mainly carried out analyses of KPNA1 expression in cervical tumor tissues and cell line.

Higher expression of KPNA2 was found in cervical cancer tissues compared with that in normal samples. This is consistent with other previous studies. Interestingly, the expression of KPNA1, another member of the karyopherin family, did not exhibit the same trend in alteration. Conversely, the KPNA1 level was downregulated in cervical tumor tissue. We also found that the proportion of cells with KPNA1-positive staining was considerably lower, and the intensity of the staining was markedly weaker in the cervical cancer cases than in the normal controls. Moreover, variations in the extent and intensity of KPNA1-positive staining were observed within different histopathologic grades of cervical cancer, ranging from weak to strong reactivity. Notably, KPNA1 expression was observed in a lower proportion of the Grade III samples than the samples of other grades, but stronger staining was observed for Grade III. To the best of our knowledge, this study is the first to report the downregulated expression of KPNA1 in cancer tumor tissue and suggests that low expression of KPNA1 may be associated with a greater degree of cervical cancer malignancy. In addition, we observed similarly low expression of KPNA1 in Hela cells compared to that in normal cervical epithelial cells. Kohler, et al. suggest that each karyopherin α family member has different substrate specificities (26). This may be the reason for the opposite trends in KPNA1 and KPNA2 expression in the same tumor cell line. Our research reveals that KPNA1 has potential for use as a diagnostic biomarker in aggressive ovarian carcinomas.

In recent years, there have been many studies demonstrating that the aberrant expression of KPNA2 is often associated with the progression and development of multiple tumors. Cui, et al. proved that the increased expression of KPNA2 accelerated ovarian tumor progression by targeting KIF4A signaling (27). Zeng, et al. reported that high expression of KPNA2 promoted the development of bladder cancer through interactions with CBX8 (28). Kubo, et al. found that enhanced KPNA2 expression was associated with the carcinogenesis of intraductal papillary mucinous neoplasms through the adenoma–carcinoma sequence (29). However, few studies on the relationship between the level of KPNA1 and tumorigenesis have been conducted. It has been suggested that the aberrant quantity and functioning of karyopherin family proteins can lead to uncontrolled cell proliferation (30, 31). To determine whether KPNA1 had functional relevance for cervical cancer cell growth, we overexpressed KPNA1 by transfecting the plasmid pCMVTNT-T7-KPNA1 into Hela cells. The results of the current study indicate that the upregulation of KPNA1 expression suppresses the proliferation of Hela cells. Although KPNA2 has received the most interest of late as a potential target for cancer therapy, it is still controversial as to whether the inhibition of KPNA2 has visible effects on tumor cells. Quensel, et al. found that the inhibition of KPNA2 did not block cell proliferation (32). On the other hand, it was reported that RNA-interference-mediated KPNA2 silencing completely inhibited the proliferation and migration of MCF7 tumor cells (33). This study has innovatively proposed that the enhancement of the KPNA1 level inhibits cervical cancer cell growth. It suggests that KPNA1 may be a potential therapeutic target for cervical cancer patients, although this remains need to be supported by more evidence. In our another paper that has been published recently, we have clarified that E6 protein induced by HPV16/18 could significantly promoted the ubiquitination and degradation of KPNA1 that resulted in the suppression of nuclear transport of phosphorylated STAT1 and apoptosis in cervical cancer cells (34). Here, we found the direct effect of KPNA1 on cervical cancer cell proliferation, which is related to transporting IRF3 to the nucleus. Moreover, the deeper mechanism by which KPNA1 inhibits tumor cell proliferation needs to be further studied in the future. Other studies have shown that the karyopherins are potential therapeutic targets for a variety of diseases such as tumor (35, 36). In particular, the importin alpha/beta-specific inhibitor has been shown to affect nuclear location of the important initiator in tumorigenesis (37). Therefore, inhibition of KPNA2-mediated excessive nuclear transport of oncoproteins by specific inhibitor may be a new treatment strategy for patients with cervical cancer.

In conclusion, this study shows that the expression of KPNA1 is decreased in cervical cancer tissues and cells. The level of KPNA1 is further decreased with an increase in the degree of cervical cancer malignancy. This is in contrast to the trend of KPNA2 expression in tumor tissues. In addition, the up-regulation of KPNA1 clearly increases the level of nuclear IRF3 and suppresses the proliferation of cervical cancer cells. Our findings suggest that KPNA1 may potentially serve as both a biomarker and therapeutic target for patients with cervical cancer.

## Data Availability

The original contributions presented in the study are included in the article/Supplementary Material, further inquiries can be directed to the corresponding authors.
