# Supplementary material for: Decreased Expression of Karyopherin-α 1 is Related to the Malignant Degree of Cervical Cancer and is Critical for the Proliferation of Hela Cells
Source: Pathol Oncol Res. 2022 Aug 4;28:1610518. doi: 10.3389/pore.2022.1610518 (PMC9385962; doi:10.3389/pore.2022.1610518)
Supplement: Supplementary file 1 [file DataSheet1.doc]

**Supplementary Materials**


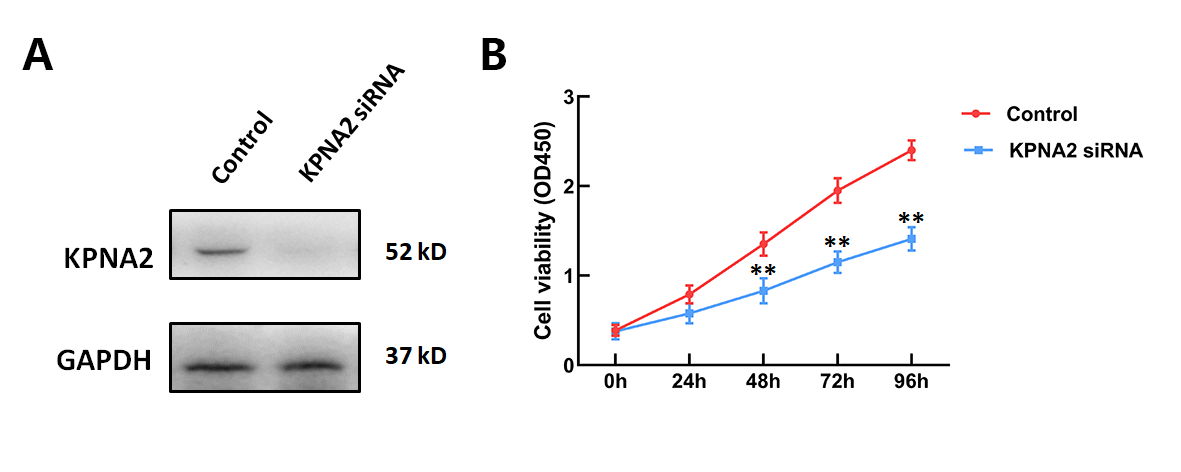


**Figure S1 The down-regulation of KPNA2 inhibited the proliferation of Hela cells.** Hela cells were transfected with control and KPNA2 siRNA plasmids (sc-35741, Santa Cruz Biotechnology, CA, USA) separately. (A) The level of KPNA2 was detected by Western blot. (B) Hela cell growth was measured by CCK-8 assay. n = 3. ***P* < 0.01 vs. the control group.
